# Black ant stings caused by *Pachycondyla sennaarensis*: a significant health hazard

**DOI:** 10.5144/0256-4947.2009.207

**Published:** 2009

**Authors:** Marzouqah AlAnazi, Mohammad AlAshahrani, Majid AlSalamah

**Affiliations:** From the Department of Emergency Medicine, King Abdulaziz Medical City-National Guard Health Affairs, Riyadh, Saudi Arabia

## Abstract

Several species of ants cause stings, but not all lead to allergic reactions. We present a series of cases of allergic reactions following insect bites or stings that presented to our emergency department and that were caused by the black samsum ant (*Pachycondyla sennaarensis*). Reactions ranged from mild allergic reactions to severe anaphylactic shock. Patients were treated with subcutaneous epinephrine 0.3 mg, intravenous methylprednisolone 125 mg, intravenous diphenhydramine HCl 50 mg, and intravenous normal saline as appropriate. These cases illustrate the range of clinical presentations to black ant stings, which can include severe reactions, indicating that ant stings are a significant public health hazard in Saudi Arabia. Physicians in the Middle East and Asia need to be aware of ant stings as a cause of severe allergic reactions.

Allergic reactions, ranging from mild reaction hives anaphylaxis and shock, are common presenting problems to emergency departments a well known medical problem facing physicians. Causes have been attributed to drugs, food, contrast agents, exercise and stress, and stings from bees, wasps and ants.^1^ Several species of ants are capable of stinging, but not all lead to allergic reactions. Fire ants have been studied thoroughly and are considered responsible for most of allergic reactions secondary to ants in the United States.^2^ Systemic reactions with fire ant stings occur in about 4 of 100 000 exposed individuals, and more than 80 fatalities have been reported from fire ant-induced anaphylaxis.^2^ In China and Korea another species of ant known as *Bachycondayla chinensis* is a major cause of allergic reactions. In addition, *Myrmecia pilosula* (jack jumper ant) in Australia is a known health hazard.^1^–^3^

We have noted a significant number of unidentified insect exposures in our emergency department that have led to different degrees of allergies. Therefore, we decided to identify the cause of these stings or bites by obtaining samples of the causal agent for identification. In this study we selected a case series of patients that were able to identify the cause of the sting as a black ant. The black ant specimens were selected carefully for photography and identification. The identification was done by a certified entomologist in the Department of Zoology, College of Science, King Saud University in Riyadh. All the specimens were collected from the eastern region of Riyadh in year 2006-2007. The species was then identified and its characteristics were described. The cases we report are cases that we encountered during our practice, ranging from mild to severe allergic reaction secondary to these black ant bites (Table 1). We note that the cases ranged from mild to severe allergic reaction requiring epinephrine. We also present a suggested treatment and follow-up algorithm.

**Table 1:** Clinical summary of cases presenting with allergy secondary to the sting of the *Pachycondyla sennaarensis*.

Case	1	2	3	4
**Demographics**	34 y/o male	27 y/o Saudi female	27 y/o Saudi female	49 y/o Saudi female with liver cirrhosis

**Presentation**	Itchy rash	Itchy rash, lightheadedness	Shortness of breath, lightheadedness, itchy rash	Shortness of breath, swelling of upper airway

**Onset of symptoms after sting**	30 minutes	Few minutes	Few minutes	10 minutes

**Vital signs**	BP: 120/80 mm Hg	BP: 105/75 mm Hg	BP: 130/85 mm Hg	BP: 95/65 mm Hg
	HR: 80/minute	HR: 99/minute	HR: 105/minute	HR: 110/minute
	RR: 18/minute	RR: 22/minute	RR: 24/minute	RR: 28/minute
	Temp: 37°c	Temp: 36.6°c	Temp: 36°c	Temp: 37°c
	O_2_ sat: 99% on RA	O_2_ sat: 98% on RA	O_2_ sat: 98% on RA	O_2_ sat: 99% on RA
		BP dropped to 77/44 after 30min		

**Physical examination**	Airway: Patent	Airway: Patent	Airway: Patent	Airway: Angioedema
	Chest: Clear	Chest: Clear	Chest: Clear	Chest: Wheezy chest
	Skin: Urticarial rash spread over limbs and trunk	Skin: Urticarial rash spread over limbs and abdomen	Skin: generalized urticaria	Skin: clear
				level of consciousness deteriorated.

**History of allergies**	No past history of allergy	No past history of allergy	Allergic rhinitis	Black ant (6th presentation with anaphylaxis)

**Causative agent**	Black ant brought by patient for identification	Black ant brought by friends for identification	Black ant brought by patient for identification	Black ant brought by family for identification

**Management**	Antihistamine, systemic steroids	Subcutaneous epinephrine injection, intravenous fluids, antihistamine, systemic steroids	Subcutaneous epinephrine injection, intravenous fluids, antihistamine, systemic steroids	Endotrachial intubation, epinephrine infusion, antihistamine, systemic steroids

**Disposition**	4 hours observation	4 hours observation	6 hours observation	ICU admission

**Discharge treatment**	Antihistamine, instructions to avoid black ant exposure	Antihistamine, instructions to avoid black ant exposure, follow up primary health care	Antihistamine, instructions to avoid black ant exposure, follow up primary health care	Epipen kit, antihistamine and instructed to eradicate black ants around her house and surroundings, and not to walk barefoot, follow-up immunology clinic

## CASE 1

A 34-year-old Saudi male presented to the emergency department complaining of generalized body rash and itching starting 30 minutes after being stung by a black ant at home. He had no other complaints such as shortness of breath, lightheadedness, chest pain or palpitations. He had no previous medical or surgical history and was not known to be allergic to any medication, food or any other allergen and never had an allergic reaction in the past. On examination he displayed normal vital signs and had a pruritic, urticarial rash over the four limbs and trunk. The patient was given methyl-prednisolone 125 mg and diphenhydramine HCI 50 mg intravenously and kept in the ER observation area for four hours until his symptoms started to subside and then he was discharged home with instructions on how to avoid future ant bites.

## CASE 2

A 27-year-old Saudi female presented one hour after being stung by a black ant at her neighbor's home. She complained of an itchy rash and lightheadedness. These complaints started minutes after being stung. She had no medical history or allergic reactions in the past. Her initial vital signs were a blood pressure of 105/75 mm Hg, a heart rate of 99 per minute, a respiratory rate of 22 per minute, a temperature of 36.6°C and an oxygen saturation of 98% on room air. After 15 minutes of presentation the patient complained of dizziness and her blood pressure dropped to 77/44 mm Hg. The patient had a generalized urticarial rash mainly over the upper limbs and abdomen. The rest of her physical examination was unremarkable. She was managed by 0.3mg subcutaneous epinephrine and a bolus of 1 liter of normal saline after which her blood pressure returned to normal. She was given methylprednisolone 125 mg and diphenhydramine HCI 50 mg intravenously and was observed in the emergency department for four hours and then discharged home in stable condition.

## CASE 3

A 27-year-old Saudi female presented to our emergency department with a chief complaint of breathlessness, dizziness and itching and redness over the body after an ant sting 2 hours prior to presentation. Symptoms started about 5 minutes after the sting. Her medical history included allergic rhinitis. Physical examination showed an anxious and restless woman with stable vital signs and generalized urticaria (Table 1). She was managed by intravenous fluid therapy, subcutaneous epinephrine injection, methylprednisolone 125 mg and diphenhydramine HCI 50 mg intravenously. After observing her for six hours, she was discharged in good condition.

## CASE 4

A 49-year-old Saudi female presented to our department after a black ant sting, complaining of shortness of breath and generalized swelling, mainly of the face and lips along with an itchy rash that started 10 minutes after being stung by a black ant at her home. This was the sixth presentation for this patient to our department suffering from anaphylaxis secondary to a black ant stings. Her medical history included a cirrhotic liver secondary to hepatits C virus infection. Initial vital signs were normal with wheezing evident on chest auscultation (Table 1). However, the patient became more distressed and the swelling increased and her level of consciousness deteriorated rapidly, so the patient underwent endotracheal intubation. The team intubating her faced a difficult intubation. They only managed to insert a 6.5 mm endotracheal tube due to airway edema. She was started on epinephrine infusion, methylprednisolone 125 mg and diphenhydramine HCI 50 mg intravenously. She was admitted to the intensive care unit for two days, and after extubation she was observed on the floor for another two days. She was discharged with an EpiPen kit and strongly advised not to walk barefoot and to perform pest control and eradication in her house and its surroundings.

## DISCUSSION

*Pachycondyla sennaarensis* belongs to the subfamily Ponerinae, a species that is widely distributed in tropical and subtropical regions worldwide. It is believed to be indigenous to Southeast Asia, but has been reported from all of the Arab Gulf countries including Kuwait, Qatar, the United Arab Emirates, Oman, Yemen and Saudi Arabia.^4^ In Saudi Arabia it seems to prefer areas of human habitation. Although the ant is a scavenger in nature, it stings human beings as a defensive behavior.^5^ Recently, several anaphylactic cases following black (samsum) ant stings were reported in local clinics in Saudi Arabia, some of them critical.^6^ This ant has been observed around and inside houses and in hospital residence areas in many locations in the Al Riyadh region. The risk to humans from a particular species probably depends on complex interactions between the likelihood of human contact, insect aggression, efficiency of the venom delivery apparatus, and venom allergenicity.

**Figure 1:**
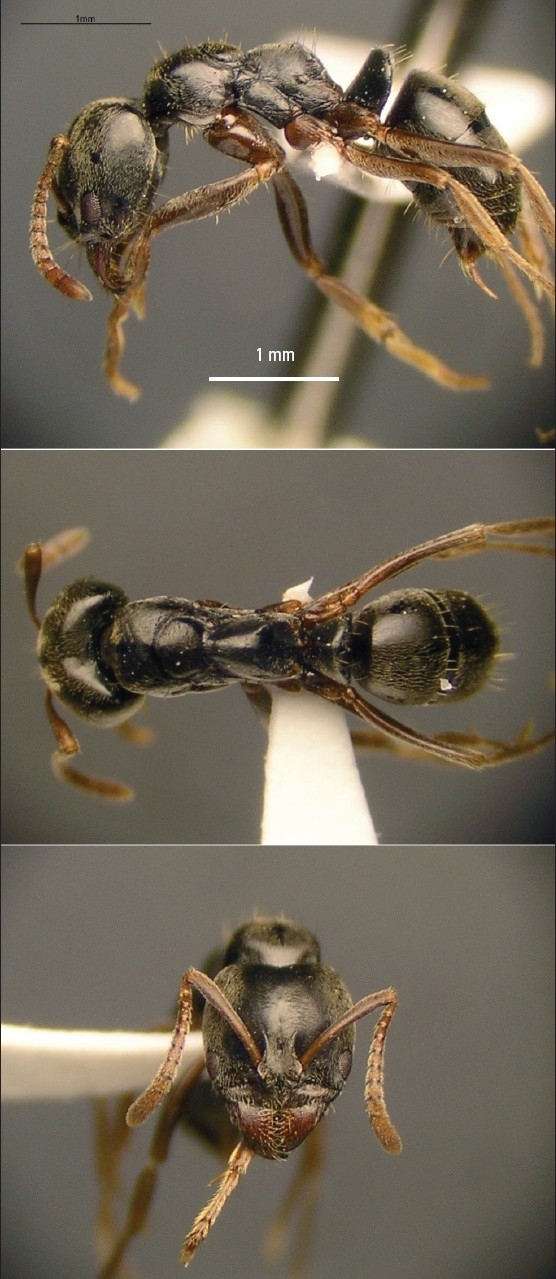
Photograph of *Pachycondyla sennaarensis* (black samsum ant). Used with permission of Mike J. Lush, sifolinia. blogspot.com, 2009.

**Figure 2:**
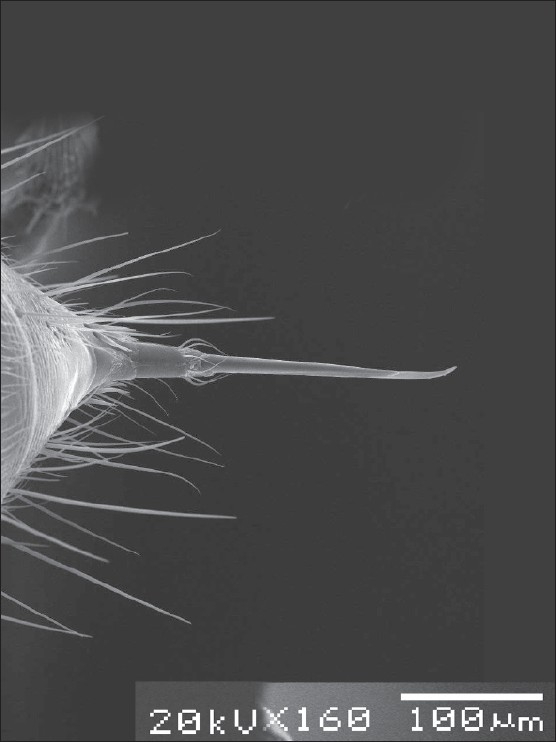
Electron microscope image of the stinger of *Pachycondyla sennaarensis* which injects allergenic venom. From the Museum of Entomology in Department of Zoology, College of Science, King Saud University, Riyadh, Saudi Arabia (Courtesy of Dr. M. Khalifa).

The most important morphological features of this ant are the large eyes and mandibles with a dorsolateral pit, and the presence of a deep mesopropodal furrow (Figure 1). It does not bite; instead, it injects venom through a stinger (Figure 2), like fire ants and other Hymenoptera. The mechanism of the reaction to the sting of the black ant *Pachycondyla sennaarensis* is a type I IgE-mediated hypersensitivity and the diagnosis can be confirmed by skin tests and specific IgE determination.^7^,^8^

The imported fire ant is a significant health problem in the southern United States.^2^ The venom from fire ants contains several allergenic proteins, which can cause anaphylaxis in allergic individuals. The safety and effectiveness of several immunotherapy options have been documented for fire ant stings.^9^,^10^ However, research has shown that patients who have had an anaphylactic reaction to a *Pachycondyla* species ant might not benefit from immunotherapy with an imported fire ant extract.^10^ Immunotherapy with the extract of *Pachycondyla* species ants is expected to be highly effective. Ant venom immunotherapy is feasible and highly efficacious.^10^ However, the limited geographical distribution of each species presents a major challenge to making venom extracts available for clinical use.^12^

**Figure 3:**
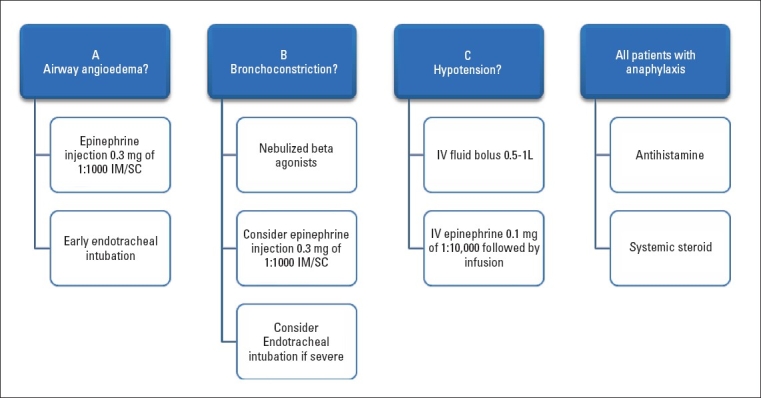
Simple algorithm for treating patients presenting with acute anaphylaxis.

In this study we confirmed the exact species, the venomous nature of its sting and the range of clinical manifestations associated with black ant stings. The acute emergency treatment depends on the manifestation that develop. The acute treatment is summarized in Figure 3. Patients need to be followed-up by AN immunologists for these cases to be managed appropriately and to document if they develop secondary reactions. The cases were referred to primary health care in this case series prior to documenting that the cause of the allergy was an ant bite.

The study of ant allergy in Asia and the Middle East is in its infancy. Clinicians in these countries need to be aware of ant stings as a cause of severe allergic reactions. Certain species that cause allergic reactions deserve further research. The allergens in the venom of the different ant species need to be identified. We should aim for improved understanding of the epidemiology of ant-sting anaphylaxis, formulation of better diagnostic tests and possibly the introduction of immunotherapeutic strategies. We believe these types of studies are required to find an effective treatment or prophylaxis against ant stings as it is a health hazard that can lead to severe morbidity and mortality. This study confirms that public health problems due to severe ant sting anaphylaxis in this region are due to the ant species *Pachycondyla sennaarensis* (samsum ant).
